# Effect of Foliar Spraying of Gibberellins and Brassinolide on Cadmium Accumulation in Rice

**DOI:** 10.3390/toxics11040364

**Published:** 2023-04-11

**Authors:** Bei Li, Song Wang, Xiaoshuang You, Zhenzhou Wen, Guirong Huang, Caicheng Huang, Qiaoxian Li, Kuiyuan Chen, Yihan Zhao, Minghua Gu, Xiaofeng Li, Yanyan Wei, Yan Qin

**Affiliations:** 1 Guangxi Key Laboratory for Agro-Environment and Agro-Products Safety, State Key Laboratory for Conservation and Utilization of Subtropical Agri-Bioresources, National Demonstration Center for Experimental Plant Science Education, College of Agriculture, Guangxi University, Nanning 530004, China; 2 Heihe Branch of Heilongjiang Academy of Agricultural Sciences, Heihe 164300, China

**Keywords:** cadmium, rice, gibberellins, brassinolide

## Abstract

Cadmium (Cd) is one of the heavy metals that contaminate rice cultivation, and reducing Cd contamination in rice through agronomic measures is a hot research topic. In this study, foliar sprays of gibberellins (GA) and brassinolide (BR) were applied to rice under Cd stress in hydroponic and pot experiments. After foliar spraying of GR and BR, the biomass of rice plants grown in either hydroponics or soil culture was significantly higher or even exceeded that in the absence of Cd stress. In addition, photosynthetic parameters (maximum fluorescence values), root length and root surface area, and CAT, SOD and POD activities were significantly improved. The MDA content decreased in the shoots, suggesting that the application of GR and BA may have enhanced photosynthesis and antioxidant function to alleviate Cd stress. Furthermore, the BR and GA treatments decreased the Cd content of rice roots, shoots and grains as well as the Cd transfer coefficient. Cd chemical morphology analysis of rice roots and shoots showed that the proportion of soluble Cd (Ethanol-Cd and Water-Cd) decreased, whereas the proportion of NaCl-Cd increased. Analysis of the subcellular distribution of Cd in rice roots and above ground showed that the proportion of Cd in the cell wall increased after foliar spraying of GA and BR. The results indicate that after foliar application of GA and BR, more of the Cd in rice was transformed into immobile forms and was fixed in the cell wall, thus reducing the amount in the seeds. In summary, foliar sprays of GA and BR can reduce the toxic effects of Cd on rice plants and reduce the Cd content in rice grains, with GA being more effective.

## 1. Introduction

Cadmium (Cd) is a heavy metal element that is harmful to human health [1]. Heavy metals are mostly present in the crust of earth in their natural condition, but excessive industrial and mining expansion as well as careless agricultural practices, have caused significant levels of Cd to contaminate soils [2]. Thus, Cd pollution is becoming a serious problem in farmlands since plants take up these elements from the soil and introduce them into the food chain, causing metal toxicity risks for humans and animals [3,4]. Rice is one of the three major staple foods globally [5]; thus, Cd contamination of rice would pose a serious threat to human health [6,7]. Cd contaminated rice poses a risk to human health so reducing the Cd content of rice is an urgent task. Many agronomic measures have been applied during crop production to decrease the crop Cd content. Different studies confirm that soil passivators have the ability to reduce the translocation of Cd from polluted soils to plants [8,9].

Recently, the exogenous addition of plant hormones has been shown to reduce heavy metal uptake in crops [10]. Phytohormones are a class of small organic molecules produced in plants, and some phytohormones (α-naphthaleneacetic acid, cytokinins, ethylene, salicylic acid, and jasmonates) participate in mediating plant responses to abiotic stress. Treatment with 0.5 μM NAA improved antioxidant enzyme activities, reduced reactive oxygen, maintained membrane permeability, and decreased malondialdehyde and proline contents. In green algae, cytokinins were found to restore the Cd-induced inhibition of photosynthetic capacity. Ethylene can reverse zinc inhibition of photosynthesis by altering the activity of photosynthetic system II, photosynthetic nitrogen use efficiency and interacting with antioxidant metabolism. Application of SA significantly improved the tolerance of gold bean and cowpea to Cd stress by increasing antioxidant enzymes and decreasing H_2_O_2_ accumulation. When formed or exogenously applied, JA decreased the Cd concentration in root cell sap by decreasing the expression of genes promoting Cd uptake and long-distance translocation, thereby attenuating Cd stress [11,12,13,14]. Many studies have shown that cytokinin, jasmonates, abscisic acid and other phytohormones respond to heavy metal stress by enhancing photosynthesis, regulating antioxidant systems and osmoregulation in plants [15]. Moreover, plant hormones are beneficial for sustainable agriculture [16]. Therefore, the application of plant hormones to reduce Cd contamination in crops and improve the quality of agricultural products has broad application prospects.

Brassinosteroids (BR), as the sixth group of plant hormones in plants, widely exist in various plant organs. Seed germination, cell division, vascular differentiation, cell elongation, senescence, flower development, male sterility, root development, stomata formation, photomorphogenesis, and resistance to various stressors including heavy metal toxicity are just a few of the activities that are regulated by BR [15]. BRs protect the Arabidopsis root system from Cd-stress by counteracting its deleterious morphogenic effects on the apices of all the root types and favoring LR and AR formation [17]. Zhong et al. found that brassinolide can improve the resistance of *Festuca californica* under lead stress by regulating the antioxidant enzyme system [18]. Bukhari et al. found that foliar applications of 24-epibrassinolide (0.1 M) on Cr-stressed tobacco significantly reduced Cr-induced inhibition of growth and photosynthesis, oxidative stress, ultrastructure damage, and Cr accumulation in different parts of leaves and roots, while maintaining ion homeostasis [19]. Gibberellin (GA) is an essential endogenous plant growth regulator that is present in the most species and has many physiological functions, including seed germination, stem elongation, leaf enlargement, blooming, and fruit formation. GA has also been linked to increased plant resistance to heavy metal stress by controlling a variety of plant activities [15]. GA3 targets shoot-root growth, morphophysiological traits, and alleviates the detrimental effects of heavy metal stress in plants [20]. Saleem et al. found that in sunflowers under Cr stress, the shoot fresh weight was reduced by 32–60% and the fresh weight of roots was reduced by 35–60%, but GA spraying significantly reduced the effect of Cr stress on plant growth. Adding 10^−4^ mol/L gibberellins (GA) could alleviate the Cr toxicity and restore the yield of sunflower [21]. According to a recent study, GA changed the amount of Cd, Cr, Cu, Ni, and Zn accumulated in the tissues and altered the development of tomato seedlings that were watered with acid mine water [22]. Based these research findings, it is possible to apply GA and BR to reduce the stress of heavy metals easily and efficiently.

There are many studies on the application of GA and BR, growth hormones that play important roles in plants. However, there are few studies on Cd uptake and transport in rice under excessive Cd. Therefore, in this study we conducted pot and hydroponic experiments with rice seedlings treated with excessive Cd as well as exogenous GA and BR treatments of the same concentration. We aimed to determine the physiological and biochemical performance of rice under Cd stress and to explore the effects of foliar spraying of gibberellin and brassinolide on Cd accumulation in rice.

## 2. Materials and Methods

### 2.1. Experimental Design

#### 2.1.1. Hydroponic Experiment

The tested rice was Y-liangyou 2, a two-line indica hybrid rice variety. The hydroponic experiment was conducted using four treatments with 3 replicates each, totaling 16 pots. The treatments were as follows: (1) control (CK): Ck was cultured in a cadmium-free Kimura B and the foliage was sprayed with deionised water; (2) Cd: The treatment with Cd alone was a culture with a Kimura B nutrient solution containing Cd (20 mol/L CdCl_2_) and a foliar spray of deionised water; (3) Cd + GA: Cd treatment + gibberellins (GA) treatment: The nutrient solution was mixed with 20 mol/L Cd (CdCl_2_) and the leaves were sprayed with 0.1 mM gibberellins (Solarbio, Beijing); (4) Cd + BR: Cd treatment + brassinolide (BR) treatment: The nutrient solution was mixed with 20 mol/L Cd (CdCl_2_) and the leaves were sprayed with 0.1 mM brassinolide (Solarbio, Beijing). The plants were cultivated in chambers with the following conditions during the plant growth period: day/night temperatures of 28 °C/22 °C; 14 h/10 h day/night cycle; and 1600 Lux.

The rice was grown in a medium consisting of the following components (mmol/L): NH_4_NO_3_ (1.45), NaH_2_PO_4_ (0.32), K_2_SO_4_ (0.5), CaCl_2_ (1.0), MgSO_4_·7H_2_O (1.7), MnCl_2_·4H_2_O (9.1 × 10^−3^), (NH_4_)_6_MoO_24_·4H_2_O (5.2 × 10^−4^), H_3_BO_3_ (1.8 × 10^−2^), ZnSO_4_·7H_2_O (1.5 × 10^−4^), CuSO_4_·5H_2_O (1.6 × 10^−4^), and FeCl_2_·6H_2_O (3.6 × 10^−2^). The pH was adjusted with HCl or NaOH to 5.5~5.8. Samples were collected after growing rice seedlings for 15 days. The shoots and roots of the rice seedlings were divided into two parts, and one part was frozen in liquid nitrogen and stored at −80 °C for the determination of antioxidant enzymes. The remaining part was oven dried at 105 °C for 30 min to a constant weight at 65 °C. The dried root and shoot samples were crushed into powder and stored for Cd testing.

#### 2.1.2. Pot Experiment

The Cd-contaminated soil was collected from Nandan County, Guangxi, China. The basic physical and chemical properties of the soil were as follows: pH value of 7.4, soil organic matter of 20.5 mg/kg, alkali-hydrolyzable nitrogen of 68 mg/kg, available phosphorus of 29.7 mg/kg, available potassium of 265 mg/kg, and Cd content of 1.62 mg/kg. Soil pH determination was performed using a portable pH meter (PH3000, Multiplex 3000 WTW, Weilheim, Germany). The physical and chemical properties of the soil were determined according to previously described methods [23,24]. The soil was air-dried, screened with a 2 mm sieve, and distributed into basins, each containing 6.5 kg of the soil. Rice seedlings with the same growth status were selected and transplanted into the soil, with three holes per pot and two seedlings per hole.

The pot experiments were carried out outdoors under natural light conditions in March, in Nanning, Guangxi. During the growth period of rice, fertilization was conducted at the normal application rate, which included nitrogen (urea) at 475 mg/kg, phosphate (potassium dihydrogen phosphate) at 750 mg/kg and potassium (potassium chloride) at 400 mg/kg. The ratio of basal to top dressing fertilizer was 7:3. There were three treatments with three replicates each, totaling nine pots. The treatments were as follows: (1) Cd: water sprayed on the foliage; (2) Cd + GA: 0.1 mM GA application on leaf surface; (3) Cd + BR: 0.1 mM BR application on the leaf surface. GA and BR were sprayed once at the rice elongation, flowering, and grain filling stages.

Samples were harvested when the rice reached maturity at 135 days, and the fresh samples from each treatment were used to determine the chemical morphology and subcellular distribution of Cd. The whole rice plant was divided into roots, shoots and grains. The roots and shoots were cured in an oven at 105 °C for 30 min and then dried to constant weight at 65 °C, and the seeds were air-dried. The dry weight of the four parts was then weighed. Subsequently, the seeds were threshed using a thresher, and the other parts were crushed using a powder machine. The samples were stored in self-sealing bags until Cd content determination.

### 2.2. Measurement Equipment and Methods

#### 2.2.1. Root Index Determination

For the hydroponic experiment, five rice seedlings were randomly selected from each treatment and washed with deionized water 3–5 times. The seedlings were then blot dried with filter paper, and a roots scanner (Epson Expression 10000XL, Epson, Suwa, Japan) was used to measure the length and surface area of the roots.

#### 2.2.2. Determination of Maximum Fluorescence Value of Leaves

The rice seedlings in the hydroponic experiment were kept in the dark for 30 min a day before leaf sample collection. The fluorescence value of the sampled fresh leaves was then measured by a portable chlorophyll fluorescence instrument (PAM-2500, Heinz Walz, Germany).

#### 2.2.3. Determination of Antioxidant Enzymes Activities

The activities of superoxide dismutase (SOD), peroxidase (POD), catalase (CAT) and the content of malondialdehyde (MDA) were determined using the following kits from the Nanjing Jiancheng Institute of Biological Engineering: A001-3-2 Superoxide Dismutase (SOD) assay kit (WST-1 method), A007-1-1 Catalase (CAT) assay kit (Visible light), A084-3-1 Peroxidase assay kit, and the A003-3-1 Plant Malondialdehyde (MDA) assay kit (Colorimetric method).

#### 2.2.4. Determination of Cd Content

A 0.1 g sample was weighed into a Teflon digestion tube and 4 mL of nitric acid (HNO_3_) and 1 mL of hydrogen peroxide (H_2_O_2_) were added for microwave digestion. Thereafter, the mixture was heated to 130 °C for 2 h to remove the acid and transferred into a 25 mL volumetric flask after cooling to room temperature. The Cd content was then determined by inductively coupled plasma mass spectrometry (ICP-MS) (NEXION 350X, PerkinElmerTM Life Science Incorporated, Waltham, MA, USA PE).

#### 2.2.5. Photosynthesis Determination in Mature Rice

Photosynthetic parameters were determined using a portable photosynthesis meter (LI-6400XT, Li-Cor, Lincoln, USA) at the last day. Briefly, the rice leaf blade was collected from each replicate, and the gas exchange parameters (in the middle part of the rice leaf) were measured in the leaf chamber with red and blue light sources. The measurement was conducted thrice at 9 a.m. when there was sufficient light, and the average value was obtained. The determination conditions were a flow meter of 500 μmol/m^2^·s and light intensity of 1500 μmol/m^2^·s. The net photosynthetic rate (Pn) and transpiration rate (Tr) were also measured.

#### 2.2.6. Determination of Chemical Morphology of Cd in Rice Plants

Different chemical forms of Cd were extracted using: (1) 80% anhydrous ethanol (denoted as Ethanol-Cd, ethanol extractable Cd); (2) water (denoted as Water-Cd, deionized water extractable Cd); (3) 1 mol/L NaCl (denoted as NaCl-Cd, sodium chloride extractable Cd); (4) 2% acetic acid (denoted as Acetic acid-Cd, acetic acid extractable Cd); (5) 0.6% mol/L HCl (denoted as HCl-Cd, hydrochloric acid extractable Cd); (6) residue (denoted as Residual-Cd). Briefly, the specific chemical extracts were added to the homogenate of rice samples (0.5 g) and diluted with water (ratio 1:100, mass to volume ratio), followed by constant temperature oscillation for 22 h (at 25 °C and 200 r/min). Thereafter, the mixture was subjected to high-speed refrigerated centrifugation (4 °C, 5000 r/min) for 10 min to collect the supernatant. The centrifugation was conducted thrice, and the obtained supernatants were mixed. The extraction steps of the five Cd states were the same. After acid digestion, the supernatant and residual hydrochloric acid extractable Cd were mixed with HNO_3_:HClO_4_ (2:1 by volume ratio). The Cd content in each part was then determined by ICP-MS.

#### 2.2.7. Determination of Subcellular Cd Content in Rice

Briefly, 1.0 g of fresh sample was weighed into a 50 mL centrifuge tube, and 15 mL of pre-cooled buffer solution (0.25 mol/L sucrose, 1 mmol/L erythritol disulfide, 50 mmol·L^−1^ TriS-HCl (pH 7.5)) was added. The mixture was then homogenized, and the different cellular components in each organ were separated by differential centrifugation. The homogenate was centrifuged for 15 min by a highspeed refrigerated centrifuge (4 °C, 3000 r/min) to precipitate the cell wall component. After discarding the supernatant, the same volume of the buffer was added, and the mixture was centrifuged for 30 min in a high-speed refrigerated centrifuge (4 °C, 3000 r/min). The obtained supernatant was the cell fluid component, while the precipitate was the organelle component. The components were oven-dried at 65 °C and then digested with a mixture of HNO_3_:HClO_4_ (volume ratio 5:1). The Cd contents of cell wall, cytosol, and organelle components were determined by ICP-MS.

#### 2.2.8. Calculation of Cd Transfer Coefficient


$$\mathrm{The transfer coefficient of roots to shoots}=\frac{\left(\mathrm{The Cd content of shoots}\right)}{\left(\mathrm{The Cd content of roots}\right)}.$$

$$\mathrm{The transfer coefficient of shoots to grains}=\frac{\left(\mathrm{The Cd content of grains}\right)}{\left(\mathrm{The Cd content of shoots}\right)}.$$

### 2.3. Data Analysis

All the experimental data were processed by Microsoft Excel 2010 and analyzed using the statistical software SPSS 21.0. Duncan’s test was used to test the significant differences between the treatments, and graphs were generated using GraphPad Prism 7.

## 3. Results

### 3.1. Effects of Foliage Spray Application of GA and BR on Growth and Cd Uptake of Rice Seedlings under Cd Stress

#### 3.1.1. Effects of Foliage Spray Application of GA and BR on Growth of Rice Seedlings under Cd Stress

As shown in Table 1, when Cd was added to the nutrient solution, the root and shoot biomass of the rice seedlings was significantly lower compared with CK, but after foliar application of GA and BR, the root and shoot biomass even exceeded that of CK and reached a significant level (*p* < 0.05).

#### 3.1.2. Effects of Foliage Spray Application of GA and BR on Root Morphology of Rice Seedings under Cd Stress

**Figure 1:**
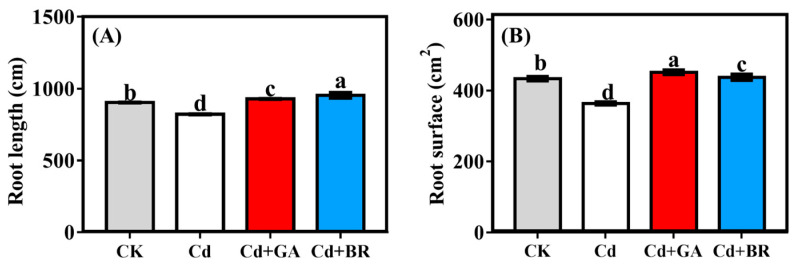
Effects of foliar application of GA and BR on the root lengths (**A**) and root surface area (**B**) of rice seedlings under Cd stress. CK indicates cultured in a cadmium-free and foliage sprayed with deionised water; Cd indicates culture with solution containing 20 mol/L CdCl_2_ and a foliar spray of deionised water; Cd + GA indicates a culture with 20 mol/L Cd (CdCl_2_) and leaves sprayed with 0.1 mM gibberellins; and Cd + BR indicates a culture with 20 mol/L Cd (CdCl_2_) and leaves sprayed with 0.1 mM brassinolide. Error bars indicate standard errors of the mean (n = 3), and the different lowercase letters indicate significant differences (*p* < 0.05) among different treatments.

After foliar spraying, the root length and root surface of rice seedlings sprayed with GA increased by 2.74% and 4.17%, respectively, compared with CK, and the root length and root surface of the rice seedlings sprayed with BR increased by 5.53% and 0.92%, respectively, compared with CK. Similar to biomass, the root length (Figure 1A) and root surface (Figure 1B) of rice decreased significantly under Cd stress.

#### 3.1.3. Effects of Foliar Application of GA and BR on Maximum Fluorescence Value of Rice Leaves under Cd Stress

**Figure 2:**
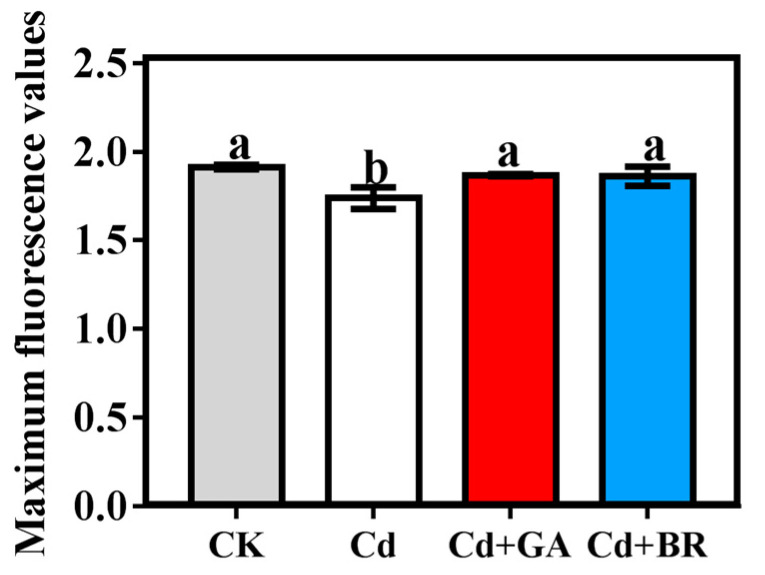
Effects of foliar application of GA and BR on the maximum fluorescence values of rice seedlings under Cd stress. Cd indicates a culture with a solution containing 20 mol/L CdCl_2_ and a foliar spray of deionised water; Cd + GA indicates a culture with 20 mol/L Cd (CdCl_2_) and leaves sprayed with 0.1 mM gibberellins; and Cd + BR indicates a culture with 20 mol/L Cd (CdCl_2_) and leaves sprayed with 0.1 mM brassinolide. Error bars indicate standard errors of mean (n = 3), and the different lowercase letters indicate significant differences (*p* < 0.05) among different treatments.

The ratio of variable fluorescence to maximum fluorescence (Fv/Fm) reflects the maximum light energy conversion efficiency of the ΦPS II reaction center. As shown in Figure 2, the maximum fluorescence value of the rice seedlings showed a Cd + GA = Cd + BR = CK > Cd trend. Cd application also reduced photosynthesis in rice leaves, and the maximum fluorescence values were restored after foliar sprays of GA and BR.

#### 3.1.4. Effects of Foliar Application of GA and BR on SOD, POD, CAT and MDA Content in Rice Seedlings under Cd Stress

**Figure 3:**
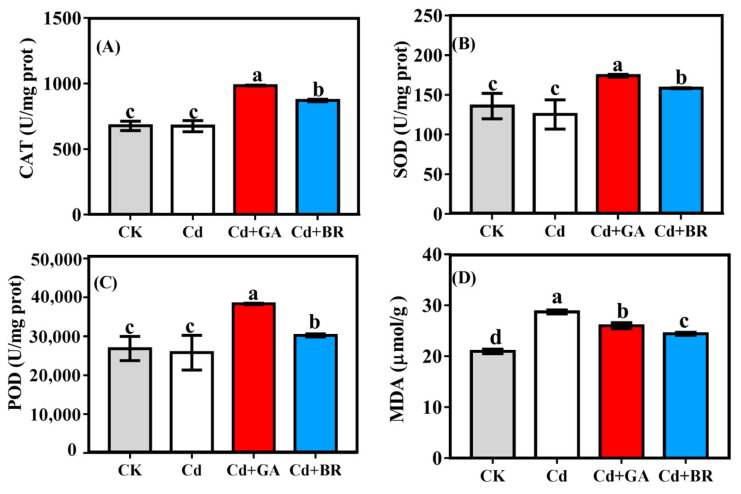
Effects of GA and BR foliar application on the activity of rice antioxidant enzymes under Cd stress. Cd indicates culture with solution containing 20 mol/L CdCl_2_ and a foliar spray of deionised water; Cd + GA indicates a culture with 20 mol/L Cd (CdCl_2_) and leaves sprayed with 0.1 mM gibberellins; Cd + BR indicates a culture with 20 mol/L Cd (CdCl_2_) and leaves sprayed with 0.1 mM brassinolide. Error bars indicate standard errors of the mean (n = 3). The effects of GA and BR foliar application on (**A**) catalase (CAT), (**B**) superoxide dismutase (SOD), (**C**) peroxidase (POD), and (**D**) malondialdehyde (MDA) activities in rice under Cd stress.

After foliar spraying with GA, the activities of CAT, SOD and POD were significantly increased, exceeding those of CK by 45.28%, 28.17% and 42.69%, respectively. After foliar spraying with BR, the activities of CAT, SOD and POD exceeded those of CK by 28.56%, 28.17% and 12.56%, respectively. While the MDA of rice seedlings under Cd stress was significantly increased under Cd stress, it was also significantly reduced by 9.5% and 14.96% after foliar spraying of GA and BR compared with the Cd group, although it did not recover to the level of CK (Figure 3D), The activities of CAT (Figure 3A), SOD (Figure 3B) and POD (Figure 3C) in rice under Cd stress were all slightly decreased relative to CK.

#### 3.1.5. Effects of Foliage Spray Application of GA and BR on Cd Content and Translocation Coefficient of Rice Seedlings under Cd Stress

**Figure 4:**
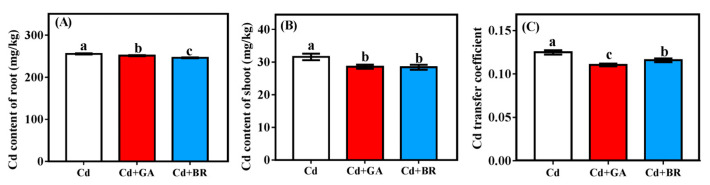
Effects of GA and BR foliar application on Cd concentration of rice seedlings under Cd stress. The effects of GA and BR foliar application on the Cd content of the (**A**) roots and (**B**) shoots of rice seedlings under Cd stress. (**C**) Effects of foliar-applied GA and BR on the Cd migration coefficient of rice seedlings. Cd indicates cultured in soil and foliage sprayed with deionised water, Cd + GA indicates 0.1 mM GA application on leaf surface, Cd + BR indicates 0.1 mM BR application on the leaf surface. Error bars indicate standard errors of the mean (n = 3), and different lowercase letters indicate significant differences (*p* < 0.05) among different treatments.

Foliar applications of both GA and BR significantly reduced Cd levels in the roots and shoots of rice seedlings (Figure 4A,B). For the roots (Figure 4A), Cd levels were equivalently lower with BR application (significantly lower than for CK and the GA application, *p* < 0.05). However, the Cd levels of the shoots after BR application were not significantly different from those with GA application (Figure 4B), so that the upward transport of Cd in rice seedlings was lower with GA application (Figure 4C).

### 3.2. Effects of Foliage Spray Application of GA and BR on Migration and Accumulation of Cd in Rice under Cd Stress

#### 3.2.1. Effects of Foliar Application of GA and BR on Biomass and Yield of Rice under Cd Stress

As shown in Table 2, the application of GA on the leaf surface significantly increased the root biomass, shoot biomass, and grain yield of mature rice by 40.46%, 38.52%, and 23.74%, respectively (*p* < 0.05), compared with CK. Moreover, the leaf application of BR significantly enhanced the root and shoot biomass of rice by 21.53% and 11.05%, respectively, but had no significant effect on the yield (*p* > 0.05).

#### 3.2.2. Effects of Foliar Application of GA and BR on Cd Chemical Morphology in Rice Plants under Cd Stress

**Figure 5:**
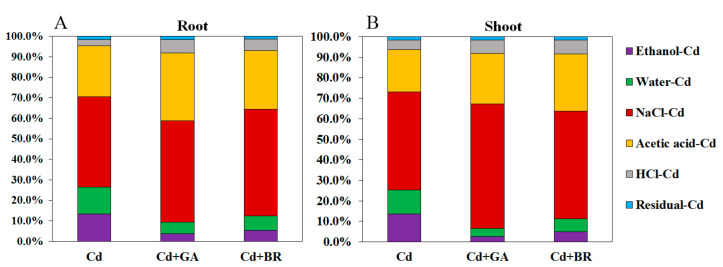
Effects of GA and BR foliar application on Cd chemical forms in rice. The effects of GA and BR foliar application on the Cd percentage in the (**A**) shoot and (**B**) root morphology of rice. Cd indicates cultured in soil and foliage is sprayed with deionised water; Cd + GA indicates a 0.1 mM GA application on leaf surface; Cd + BR indicates a 0.1 mM BR application on the leaf surface. Here, Ethanol-Cd indicates ethanol extractable Cd; Water-Cd indicates deionized water extractable Cd; NaCl-Cd indicates sodium chloride extractable Cd; Acetic acid-Cd indicates acetic acid extractable Cd; HCl-Cd indicates hydrochloric acid extractable Cd; Residual-Cd indicates residue Cd.

The results in Figure 5 show that under Cd stress, there was a more significant increase in the percentage of NaCl-Cd, HCl-Cd and Acetic acid-Cd, a significant decrease in Ethanol-Cd and Water-Cd, and a slight increase in Residual-Cd in the roots and shoots of rice seedlings after foliar spraying of GA and BR. In particular, GA application reduced the aboveground Ethanol-Cd and Water-Cd percentage and increased the NaCl-Cd percentage more than BR application.

#### 3.2.3. Effects of Foliar Application of GA and BR on Subcellular Distribution of Cd in Rice Cells under Cd Stress

**Figure 6:**
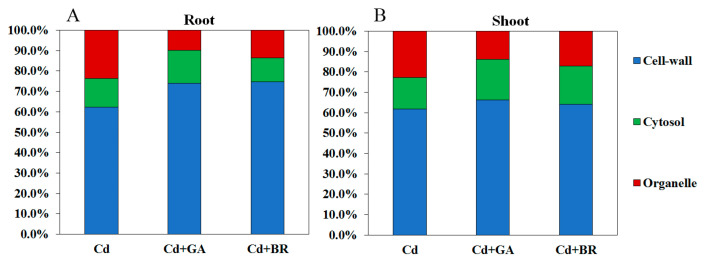
Effects of GA and BR foliar application on subcellular Cd distributions in rice (root (**A**) and shoot (**B**)) under Cd stress. Cd indicates cultured in soil and foliage sprayed with deionised water; Cd + GA indicates a 0.1 mM GA application on the leaf surface; Cd + BR indicates a 0.1 mM BR application on the leaf surface. Cell-wall indicates cell wall component; Cytosol indicates cell fluid component; Organelle indicates organelle component.

Compared with the Cd application alone, the GA treatment significantly increased the proportion of Cd in the cell wall and the cytosol by 18.77% and 15.66%, respectively, and reduced the proportion of Cd in the organelle by 58.86%. The BR treatment significantly increased the proportion of Cd in the cell wall by 20.06% and significantly reduced the proportion of Cd in the cytosol and organelles by 16.56% and 42.98%, respectively. The percentage subcellular distribution of Cd in rice shoots under GA and BR treatments showed similar trends to those of roots (Figure 6). The GA treatment increased the Cd proportion in the cell wall component by 19.41% but decreased the Cd proportion in the organelle component by 55.20%. Furthermore, the BR treatment increased the Cd proportion in the cell wall component by 14.70% but decreased the Cd content in the organelle component by 41.52%.

#### 3.2.4. Effects of Foliar Application of GA and BR on Cd Content of the Different Parts of Rice under Cd Stress

**Figure 7:**
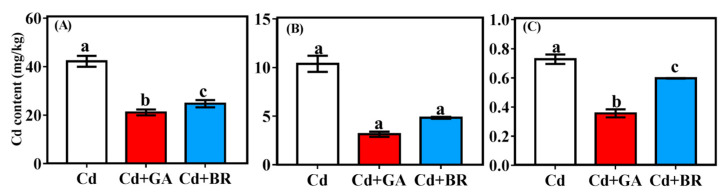
Effects of foliar application of GA and BR on Cd contents in the root, shoot and grain of rice. The effects of GA and BR foliar application on the Cd content of the (**A**) roots, (**B**) shoots, and (**C**) grains of rice under Cd stress. Cd indicates cultured in soil and foliage sprayed with deionised water; Cd + GA indicates a 0.1 mM GA application on the leaf surface; Cd + BR indicates a 0.1 mM BR application on the leaf surface. Different letters in the same list indicate significant differences (*p* < 0.05, n = 3).

Compared with the Cd treatment alone, the Cd root content of rice plants decreased by 34.42% and 23.29% under Cd + GA and Cd + BR treatments, respectively. The Cd content of the shoot decreased by 69.78% and 53.74%, respectively, while that of the grains decreased by 75.08% and 59.46% after foliar application of GA and BR, respectively (Figure 7).

#### 3.2.5. Effects of Foliar Application of GA and BR on Cd Transfer Coefficient of Rice under Cd Stress

**Figure 8:**
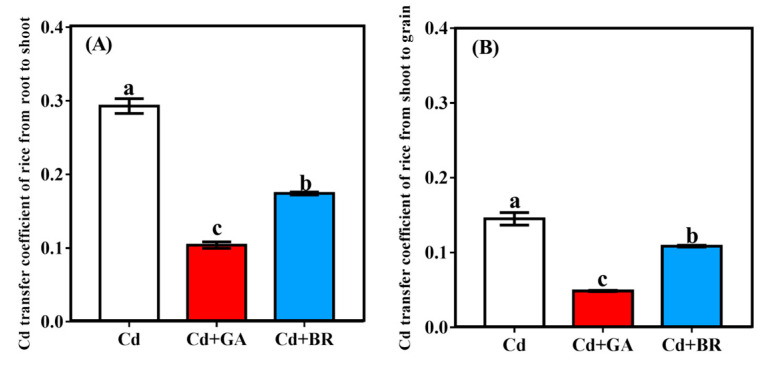
The Cd transfer coefficient in rice plants under Cd stress. (**A**) The transfer coefficient of Cd migration from the underground to the aboveground parts of rice after GA and BR foliar application; (**B**) The transfer coefficient of Cd migration from the stem to the rice grains after GA and BR foliar application. Cd indicates cultured in soil and foliage sprayed with deionised water; Cd + GA indicates a 0.1 mM GA application on the leaf surface; Cd + BR indicates 0.1 mM BR application on the leaf surface. Different letters in the same list indicate significant differences (*p* < 0.05, n = 3).

The Cd migration coefficients from the roots to the shoots decreased by 54.01% and 39.44%, respectively, while that from the shoot to grains decreased by 17.54% and 12.98% under GA and BR treatments, respectively (Figure 8). The GA treatment significantly decreased the Cd migration coefficient in rice more than the BR treatment under Cd stress. Compared with Cd treatment alone, Cd + GA and Cd + BR treatments significantly reduced the Cd migration coefficients under Cd stress.

## 4. Discussion

Cd is one of the most toxic heavy metals for plants [25]. When Cd is accumulated to a certain concentration, it retards plant growth and reduces crop yield [26]. In this study, the biomass of rice in both the hydroponics and pot experiments was decreased when Cd was the only treatment applied, indicating that Cd is toxic to rice and thus affects rice growth. In contrast, foliar sprays of GA and BR increased the biomass of Cd-stressed rice in hydroponics and pot experiments (Table 1 and Table 2). In addition, the application of GA and BR significantly increased the root length and root surface of rice seedlings under Cd stress (Figure 1), suggesting that the use of GA and BR alleviated the toxic effects of Cd on rice. This result is in accordance with previous research [27,28,29]. In this study, the photosynthesis of rice seedlings was reduced under Cd stress alone (with significantly lower maximum fluorescence values compared with CK). The maximum fluorescence values of rice were not significantly different from CK following the application of GA and BR on rice under Cd stress, indicating that photosynthesis was restored. It was observed that GA influences photosynthesis as it can modulate chloroplast development and stomata opening [30], as well as increase the chlorophyll content [31]. GA has also been found to increase net photosynthesis in leaves [32] and alter biomass production in transgenic tobacco plants [33]. Similarly, BR has been shown to increase the photosynthetic capacity of plants [34]. In addition, GA and BR have been reported to improve the antioxidant capacity of plants; specifically, they enhanced the antioxidant activities and reduced the oxidative stress in wheat and cucumber [35,36]. In the present study, a slight decrease in SOD, CAT and POD activities was observed in rice seedlings under Cd stress, while the MDA content was significantly increased. When GA and BR were applied, the antioxidant enzyme activities were increased while the MDA content was decreased, suggesting that exogenous GA and BR can improve the photosynthesis and antioxidant properties of rice and thus enhance the resistance of rice to Cd stress. However, earlier studies have shown that 100 μM of GA had the reverse effect on pea seedling growth under Cr stress [37]. It has recently been shown that mustard seedlings exhibit the best resistance to lead stress with the application of 10^−8^ M of brassinosteroids. At this concentration, the Pb content increased in seedlings without affecting seedling growth [38]. These results are quite different from our experimental results, indicating that the effects produced when applying GA and BR to crops under heavy metal stress are closely related to the species of the crop, the type of heavy metal, and the concentration of GA and BR applied. More in-depth studies are needed before applying this practice in production.

Rice grains are a major source of human Cd-intake [39]. In this study, foliar spraying of GA and BR significantly reduced the Cd content of rice roots, shoots, and grains. Additionally, the transport coefficient of Cd followed a declining trend, indicating the important role of GA and BR in the transport of Cd in rice. Different forms of Cd were extracted from the roots and aboveground parts of rice. The results showed that the application of GA and BR significantly decreased the proportion of Ethanol-Cd and Water-Cd in rice roots and shoots. This also showed that Cd-containing nitrates, chloride-based inorganic salts, amino acid salts, water-soluble organic acids, and phosphates were reduced. In contrast, the proportion of pectin- and protein-bound Cd (NaCl-Cd) increased significantly. Pectin and proteins play an important role in binding Cd in the cell walls of plants [40], enabling the cell wall to accumulate heavy metals and decrease their migration [41,42]. GA can promote plant cell wall growth and increase cell wall thickness [43]. Meanwhile, BR is a plant steroid hormone that plays an important role in regulating cell wall integrity and remodeling in plants [44]. In the present study, the application of GA and BR increased the proportion of Cd in the cell wall of rice roots and shoots, suggesting that GA and BR application can promote cell wall growth. Cd was bound and immobilized in the cell wall by pectin and protein.

Although foliar sprays of both GA and BR reduced Cd stress in rice, the application of GA was more effective. The difference in the Cd-reducing effect exhibited by the two hormones may be related to the effects on the biomass of the rice. GA application was superior to BR in promoting rice growth and yield, thus resulting in biological dilution. GA and BR also differ in their effects on the cell wall. GA affects the regulation of cell-wall loosening proteins and enzymes such as expansion and xyloglucan endotransglucosylase/hydrolase (XTH) [45], while BR is associated with the response to pectin [46]. This may explain the differences in the performance of rice against Cd stress when GA and BR were applied in this study.

## 5. Conclusions

The application of GA and BR can increase photosynthesis in rice, improve the antioxidant system and reduce oxidative damage. In addition, foliar application of GA and BR can increase rice biomass under Cd stress and alleviate the toxicity of Cd. GA and BR application reduces Cd accumulation in rice shoots and grains, mainly because more Cd is bound to pectin and protein and immobilized in the cell wall, thereby limiting its transport. In summary, GA and BR improved the ability of rice plants to cope with Cd stress and reduced the cadmium content of rice grains. Therefore, GA and BR foliar application offers a hopeful technique for the treatment of agricultural land with low to moderate heavy metal contamination because it is straightforward, inexpensive, and environmentally friendly.

## Figures and Tables

**Table 1:** Effect of foliar-applied GA and BR on biomass of rice seedlings. Different letters represent significant differences in results (*p* < 0.05, n = 3).

Treatments	Shoots DW (mg/Plant)	Roots DW (mg/Plant)
CK	566.67 ± 5.77c	214.00 ± 3.61c
Cd	463.33 ± 5.77d	157.67 ± 2.52d
Cd + GA	686.67 ± 5.77a	290.67 ± 4.04a
Cd + BR	606.67 ± 5.77b	229.67 ± 3.06b

**Table 2:** Effects of foliar application of GA and BR on rice biomass. Different letters in the same list indicate significant differences (*p* < 0.05, n = 3).

Treatments	Root Biomass (DW, g/Pot)	Shoot Biomass (DW, g/Pot)	Yield (DW, g/Pot)
Cd	6.303 ± 0.48c	34.48 ± 1.30c	73.93 ± 4.13b
Cd + GA	8.853 ± 0.51a	47.76 ± 1.75a	91.48 ± 2.82a
Cd + BR	7.660 ± 0.55b	38.29 ± 1.85b	78.29 ± 2.90b

Note: Cd indicates cultured in soil with and foliage sprayed with deionised water; Cd + GA indicates with 1 mM GA application on leaf surface; Cd + BR indicates 0.1 mM BR application on the leaf surface.

## Data Availability

Not applicable.

## References

[1] Rahimzadeh M.R., Rahimzadeh M.R., Kazemi S., Moghadamnia A.A. (2017). Cadmium toxicity and treatment: An update. Casp. J. Intern. Med..

[2] Ke S., Cheng X.Y., Zhang N., Hu H.G., Yan Q., Hou L.L., Sun X., Chen Z.N. (2015). Cadmium contamination of rice from various polluted areas of China and its potential risks to human health. Environ. Monit. Assess..

[3] Xu X., Qian J., Xie E., Shi X.Z., Zhao Y.C. (2018). Spatio-temporal change and pollution risk of agricultural soil cadmium in a rapidly industrializing area in the yangtze delta region of China. Int. J. Environ. Res..

[4] Sabella E., Luvisi A., Genga A., De Bellis L., Aprile A. (2021). Molecular responses to cadmium exposure in two contrasting durum wheat genotypes. Int. J. Mol. Sci..

[5] Milovanovic V., Smutka L. (2017). Asian countries in the global rice market. ACTA Univ. Agric. Silvic. Mendel. Brun..

[6] Wallin M., Barregard L., Sallsten G., Lundh T., Karlsson M.K., Lorentzon M., Ohlsson C., Mellström D. (2016). Low-level cadmium exposure is associated with decreased bone mineral density and increased risk of incident fractures in elderly men: The MrOS Sweden Study. J. Bone Miner. Res..

[7] Aoshima K. (2016). Itai-itai disease: Renal tubular osteomalacia induced by environmental exposure to cadmium—Historical review and perspectives. Soil Sci. Plant Nutr..

[8] Bashir S., Adeel M., Gulshan A.B., Iqbal J., Khan S., Rehman M., Azeem M. (2019). Effects of organic and inorganic passivators on the immobilization of cadmium in contaminated soils: A review. Environ. Eng. Sci..

[9] Zhang J., Tan Z., Huang Q. (2021). Study on principles and mechanisms of new biochar passivation of cadmium in soil. BioChar.

[10] Rahman S.U., Li Y., Hussain S., Hussain B., Riaz L., Ashraf M.N., Khaliq M.A., Du Z., Cheng H. (2023). Role of phytohormones in heavy metal tolerance in plants: A review. Ecol. Indic..

[11] Guan X., Sui C., Luo K., Chen Z., Feng C., Dong X., Zeng B., Dong X., Liu X. (2022). Effects of α-naphthylacetic acid on cadmium stress and related factors of tomato by regulation of gene expression. Agronomy.

[12] De Smet S., Cuypers A., Vangronsveld J., Remans T. (2015). Gene networks involved in hormonal control of root development in arabidopsis thaliana: A framework for studying its disturbance by metal stress. Int. J. Mol. Sci..

[13] Chmur M., Bajguz A. (2023). Melatonin involved in protective effects against cadmium stress in wolffia arrhizal. Int. J. Mol. Sci..

[14] Betti C., Della Rovere F., Piacentini D., Fattorini L., Falasca G., Altamura M.M. (2021). Jasmonates, ethylene and brassinosteroids control adventitious and lateral rooting as stress avoidance responses to heavy metals and metalloids. Biomolecules.

[15] Saini S., Kaur N., Pati P.K. (2021). Phytohormones: Key players in the modulation of heavy metal stress tolerance in plants. Ecotoxicol. Environ. Saf..

[16] Muhammad A.B., Qurat-Ul-Ain R., Hafiz M.A.R., Muhammad U.S., Abdur R., Kashif A.K., Muhammad I., Muhammad W., Christophe H. (2022). Key Aspects of Plant Hormones in Agricultural Sustainability under Climate Change. Plant Hormones: Recent Advances, New Perspectives and Applications.

[17] Della Rovere F., Piacentini D., Fattorini L., Girardi N., Bellanima D., Falasca G., Altamura M.M., Betti C. (2022). Brassinosteroids mitigate cadmium effects in arabidopsis root system without any cooperation with nitric oxide. Int. J. Mol. Sci..

[18] Zhong W., Xie C., Hu D., Pu S., Xiong X., Ma J., Sun L., Huang Z., Jiang M., Li X. (2020). Effect of 24-epibrassinolide on reactive oxygen species and antioxidative defense systems in tall fescue plants under lead stress. Ecotoxicol. Environ. Saf..

[19] Bukhari S.A.H., Wang R., Wang W., Ahmed I.M., Zheng W., Cao F. (2016). Genotype-dependent effect of exogenous 24-epibrassinolide on chromium-induced changes in ultrastructure and physicochemical traits in tobacco seedlings. Environ. Sci. Pollut. Res..

[20] Hakla H.R., Sharma S., Urfan M., Yadav N.S., Rajput P., Kotwal D., Pal S. (2021). Gibberellins target shoot-root growth, morpho-physiological and molecular pathways to induce cadmium tolerance in *Vigna radiata* L.. Agronomy.

[21] Saleem M., Asghar H.N., Khan M.Y., Zahir Z.A. (2015). Gibberellic acid in combination with pressmud enhances the growth of sunflower and stabilizes chromium(VI)-contaminated soil. Environ. Sci. Pollut. Res..

[22] Ogugua U.V., Kanu S.A., Ntushelo K. (2022). Gibberellic acid improves growth and reduces heavy metal accumulation: A case study in tomato (*Solanum lycopersicum* L.) seedlings exposed to acid mine water. Heliyon.

[23] Wulanningtyas H.S., Gong Y., Li P., Sakagami N., Nishiwaki J., Komatsuzaki M. (2021). A cover crop and no-tillage system for enhancing soil health by increasing soil organic matter in soybean cultivation. Soil Tillage Res..

[24] Liu Z., Li S., Liu N., Huang G., Zhou Q. (2022). Soil microbial community driven by soil moisture and nitrogen in milk vetch (*Astragalus sinicus* L.)–rapeseed (*Brassica napus* L.) intercropping. Agriculture.

[25] Zhao F.J., Wang P. (2020). Arsenic and cadmium accumulation in rice and mitigation strategies. Plant Soil.

[26] Demim S., Drouiche N., Aouabed A., Benayad T., Dendene-Badache O., Semsari S. (2013). Cadmium and nickel: Assessment of the physiological effects and heavy metal removal using a response surface approach by *L*. *gibba*. Ecol. Eng..

[27] Hadi F., Bano A., Fuller M.P. (2010). The improved phytoextraction of lead (Pb) and the growth of maize (*Zea mays* L.): The role of plant growth regulators (GA3 and IAA) and EDTA alone and in combinations. Chemosphere.

[28] Vanstraelen M., Benková E. (2012). Hormonal interactions in the regulation of plant development. Annu. Rev. Cell Dev. Biol..

[29] Robert-Seilaniantz A., Grant M., Jones J.D. (2011). Hormone crosstalk in plant disease and defense: More than just jasmonate-salicylate antagonism. Annu. Rev. Phytopathol..

[30] Müller M., Munné-Bosch S. (2021). Hormonal impact on photosynthesis and photoprotection in plants. Plant Physiol..

[31] Kadioglu A. (1992). The effects of gibberellic acid on photosynthetic pigments and oxygen evolution in Chlamydomonas and Anacystis. Biol. Plant..

[32] Iqbal N., Nazar R., Khan MI R., Masood A., Khan N.A. (2011). Role of gibberellins in regulation of source-sink relations under optimal and limiting environmental conditions. Curr. Sci..

[33] Biemelt S., Tschiersch H., Sonnewald U. (2004). Impact of altered gibberellin metabolism on biomass accumulation, lignin biosynthesis, and photosynthesis in transgenic tobacco plants. Plant Physiol..

[34] Yang P., Wang Y., Li J., Bian Z. (2019). Effects of brassinosteroids on photosynthetic performance and nitrogen metabolism in pepper seedlings under chilling stress. Agronomy.

[35] Yang P., Azher Nawaz M., Li F., Bai L., Li J. (2019). Brassinosteroids regulate antioxidant system and protect chloroplast ultrastructure of autotoxicity-stressed cucumber (*Cucumis sativus* L.) seedlings. Agronomy.

[36] Alharby H.F., Rizwan M., Iftikhar A., Hussaini K.M., ur Rehman M.Z., Bamagoos A.A., Alharbi B.M., Asrar M., Yasmeen T., Ali S. (2021). Effect of gibberellic acid and titanium dioxide nanoparticles on growth, antioxidant defense system and mineral nutrient uptake in wheat. Ecotoxicol. Environ. Saf..

[37] Gangwar S., Singh V.P., Srivastava P.K., Maurya J.N. (2011). Modification of chromium (VI) phytotoxicity by exogenous gibberellic acid application in *Pisum sativum* (L.) seedlings. Acta Physiol. Plant..

[38] Soares T.F.S.N., dos Santos Dias D.C.F., Oliveira A.M.S., Ribeiro D.M., dos Santos Dias L.A. (2020). Exogenous brassinosteroids increase lead stress tolerance in seed germination and seedling growth of *Brassica juncea* L.. Ecotoxicol. Environ. Saf..

[39] Uraguchi S., Fujiwara T. (2012). Cadmium transport and tolerance in rice: Perspectives for reducing grain cadmium accumulation. Rice.

[40] Yu H., Wu Y., Huang H., Zhan J., Wang K., Li T. (2020). The predominant role of pectin in binding Cd in the root cell wall of a high Cd accumulating rice line (*Oryza sativa* L.). Ecotoxicol. Environ. Saf..

[41] Loix C., Huybrechts M., Vangronsveld J., Gielen M., Keunen E., Cuypers A. (2017). Reciprocal interactions between Cadmium-Induced Cell Wall Responses and Oxidative Stress in Plants. Front. Plant Sci..

[42] Krzesłowska M. (2011). The cell wall in plant cell response to trace metals: Polysaccharide remodeling and its role in defense strategy. Acta Physiol. Plant..

[43] Stant M.Y. (1963). The Effect of gibberellic acid on cell width and the cell-wall of some phloem fibres. Ann. Bot..

[44] Rao X., Dixon R.A. (2017). Brassinosteroid mediated cell wall remodeling in grasses under abiotic stress. Front. Plant Sci..

[45] Xu Q., Krishnan S., Merewitz E., Xu J., Huang B. (2016). Gibberellin-regulation and genetic variations in leaf elongation for tall fescue in association with differential gene expression controlling cell expansion. Sci. Rep..

[46] Wolf S., van der Does D., Ladwig F., Sticht C., Kolbeck A., Schürholz A.K., Augustin S., Keinath N., Rausch T., Greiner S. (2014). A receptor-like protein mediates the response to pectin modification by activating brassinosteroid signaling. Proc. Natl. Acad. Sci. USA.

